# Changes in the motifs in the D0 and SD2 domains of the S protein drive the evolution of virulence in enteric coronavirus porcine epidemic diarrhea virus

**DOI:** 10.1128/jvi.02092-24

**Published:** 2025-03-04

**Authors:** Zhiqian Ma, Zhiwei Li, Yongqi Li, Xiaojing Zhao, Congsen Zheng, Yang Li, Xuyang Guo, Lele Xu, Zifang Zheng, Guangliang Liu, Haixue Zheng, Shuqi Xiao

**Affiliations:** 1 State Key Laboratory for Animal Disease Control and Prevention, Lanzhou Veterinary Research Institute, Chinese Academy of Agricultural Sciences 111658 , Lanzhou, Gansu, China; 2 College of Veterinary Medicine, Lanzhou University 12426 , Lanzhou, Gansu, China; Loyola University Chicago - Health Sciences Campus, Maywood, Illinois, USA

**Keywords:** porcine epidemic diarrhea virus, virulence evolution, spike protein, N-linked glycosylation, reverse genetic system

## Abstract

Since 2010, highly virulent mutant GII subtype porcine epidemic diarrhea virus (PEDV) strains derived from GI subtype strains have caused significant economic losses in the pig industry. However, the molecular mechanism of PEDV virulence evolution remains unclear. It has been predicted that, compared to the S proteins of GI strains, five N-linked glycosylation sites have changed in the highly virulent GII PEDV strains. To investigate how changes in these sites affect PEDV virulence, we constructed five recombinant strains harboring the above mutation sites using the GII subtype rPEDV-S_wt_ as the backbone, among which rPEDV-S_mut62_, rPEDV-S_mut118_, rPEDV-S_mut131_, and rPEDV-S_mut722_ were successfully rescued, but rPEDV-S_mut235_ was not. Compared to infection with rPEDV-S_wt_ (100%), infection with rPEDV-S_mut62_ and rPEDV-S_mut722_ resulted in lower mortality in piglets (33%), and although rPEDV-S_mut118_ and rPEDV-S_mut131_ resulted in high mortality (100%), death was delayed. All surviving piglets were challenged orally with rPEDV-S_wt_ at 21 days post-infection. The piglets in the rPEDV-S_mut62_ and rPEDV-S_mut722_ groups produced high levels of IgG, IgA, and cross-protective neutralizing antibodies, which protected the piglets after rPEDV-S_wt_ challenge. Furthermore, the change in the structures of the rPEDV-S_mut62_ and rPEDV-S_mut722_ S proteins predicted with high precision by AlphaFold 3 may be the cause of the attenuated virulence. Our data provide a unique perspective on the molecular mechanism of PEDV virulence evolution from the GI to the GII subtype and identify the targets of PED live attenuated vaccines.

**IMPORTANCE:**

The continuous emergence of novel viral variants in the current landscape poses challenges for disease prevention and control. Before 2010, PED caused by GI strains was only sporadic outbreaks and not large-scale epidemics. Since 2010, highly virulent GII strains derived from GI strains have spread worldwide and caused significant economic losses. However, the molecular mechanism underlying the differences in virulence is still unclear. In this study, the differences in the predicted glycosylation sites of the S protein between the GI and GII strains were taken as the starting point to explore the key sites responsible for the variations in PEDV virulence. The results indicate that the motifs 57ENQGVNST64 and 722NSTF725 of the S protein in the GII strains are involved in the evolution of PEDV virulence. This study provides a new perspective on the molecular mechanism of PEDV virulence evolution.

## INTRODUCTION

Porcine epidemic diarrhea (PED), caused by PED virus (PEDV), poses a threat to the development of healthy animals in the pork industry worldwide and has not been effectively controlled to date. This is mainly because the variations and pathogenic mechanisms of PEDV are not fully clear. Understanding the epidemic variation mechanism of PEDV can contribute to effectively preventing and controlling PED.

PEDV is a single-stranded, positive-sense RNA virus that belongs to the *Coronaviridae* family. The PEDV genome is approximately 28 kb in length and encodes 4 structural proteins (S, E, M, and N), 16 nonstructural proteins (nsp1–nsp16), and 1 accessory protein (ORF3) (1). The S protein is a highly glycosylated trimeric protein that plays essential roles in the variation, tissue and cell tropism, pathogenicity, and infectivity of coronaviruses (2–5). The S glycoprotein is the main virulence factor of PEDV and is composed of an N-terminal receptor-binding S1 subunit and a C-terminal S2 subunit that contains a fusion element (6). Modification of the S protein or the loss of its structural integrity, which occurs in some naturally evolved strains, affects the virulence of PEDV (7–10).

Posttranslational modifications (PTMs) of viral proteins are crucial for immune escape and pathogenicity (11). The R203K+G204R mutation increases phosphorylation of the nucleocapsid (N) protein of severe acute respiratory syndrome coronavirus 2 (SARS-CoV-2), thereby enhancing its replication and pathogenesis (12). Acetylating K389 of the NS3 helicase of flaviviruses is indispensable for virus replication and infection (13). Among the many PTMs, glycosylation plays essential roles in protein folding, structure, and function (14). Glycosylation of viral proteins affects their receptor affinity, immune evasion, and pathogenicity. Removing the N-glycosyl groups from SARS-CoV-2 spike proteins enhances the binding affinity of neutralizing antibodies for the virus and reduces virus infectivity (15–17). Two glycosylation sites in the S1^B^ domain of the porcine deltacoronavirus (PDCoV) S glycoprotein are critical for aminopeptidase N (APN) binding (18). Changes in the glycosylation sites of viral glycoproteins affect the virulence of the virus; for example, NSP4 glycosylation-defective rotavirus is less pathogenic than the wild-type virus, and unique glycosylation at position 986 on the E2 glycoprotein is responsible for viral attenuation (19, 20). However, little is known about the roles PTMs of PEDV proteins play in viral pathogenicity.

PEDV was first discovered in Belgium and the United Kingdom in 1976 and was detected in China as early as the 1980s (21). PEDV has evolved into two groups: GI (classical) and GII (variant). Furthermore, based on phylogenetic analyses of the complete genomes of PEDV, GI strains can be classified as GI‐a and GI‐b, and GII strains can be classified as GII‐a, GII‐b, and GII‐c (22). The GII‐c strains evolved from a recombinant virus that acquired the 5′ section of the spike gene from the GI‐a subgroup and the remaining genomic regions from the GII‐a subgroup. Before 2010, only sporadic outbreaks of PED caused by the GI genogroup occurred, but no large-scale epidemics occurred (22). Since 2010, PEDV variants, which are included mainly within the GII group, have spread worldwide and caused significant economic losses. GII-c strains appeared in 2010 and are intermediate strains that mutated from GI strains to GII strains. Compared to those in GI subtype strains, amino acid mutations, deletions, and insertions in the S and E proteins have been found in GII subtype strains (23, 24). Beginning in 2010, GII strains with increased virulence have abruptly emerged and spread on a large scale, but the mechanism underlying their virulence is unknown. The PEDV S protein is a key protein for strain classification. It is still unclear which motifs or sites in the PEDV S protein have an effect on the evolution of virus virulence. Owing to the many sites at which the S protein is expressed, we first focused on how the changes in the glycosylation sites of the PEDV S protein affected the evolution of virus virulence from the GI to the GII subtypes. Compared to the S proteins of PEDV GI subtype strains, five N-linked glycosylation sites with N-X-S/T motifs in the GII subtype PEDV strains, which are located at positions aa 62, aa 116, aa 131, aa 233, and aa 722, were predicted to change. We wondered whether changes in these motifs are part of the mechanism by which PEDV strains are attenuated.

In this study, a series of recombinant strains rPEDV-S_mut62_, rPEDV-S_mut118_, rPEDV-S_mut131_, and rPEDV-S_mut722_ were obtained using the highly virulent GII rPEDV-S_wt_ strain as the backbone. Compared to that of the rPEDV-S_wt_ strain, the virulence of the rPEDV-S_mut62_ and rPEDV-S_mut722_ strains was mildly attenuated. In addition, the rPEDV-S_mut62_ and rPEDV-S_mut722_ retained immunogenicity and protected pigs from challenge with parental PEDV. This work indicates that the motifs ^57^ENQGVNST^64^ and ^722^SSTF^725^ of the S protein in GII strains participate in the evolution of PEDV virulence, which provides a new perspective on the molecular mechanism of PEDV virulence evolution and the targets of live attenuated PED vaccines.

## RESULTS

### The S protein mutation sites during PEDV evolution from the GI to GII strains

**Fig 1:**
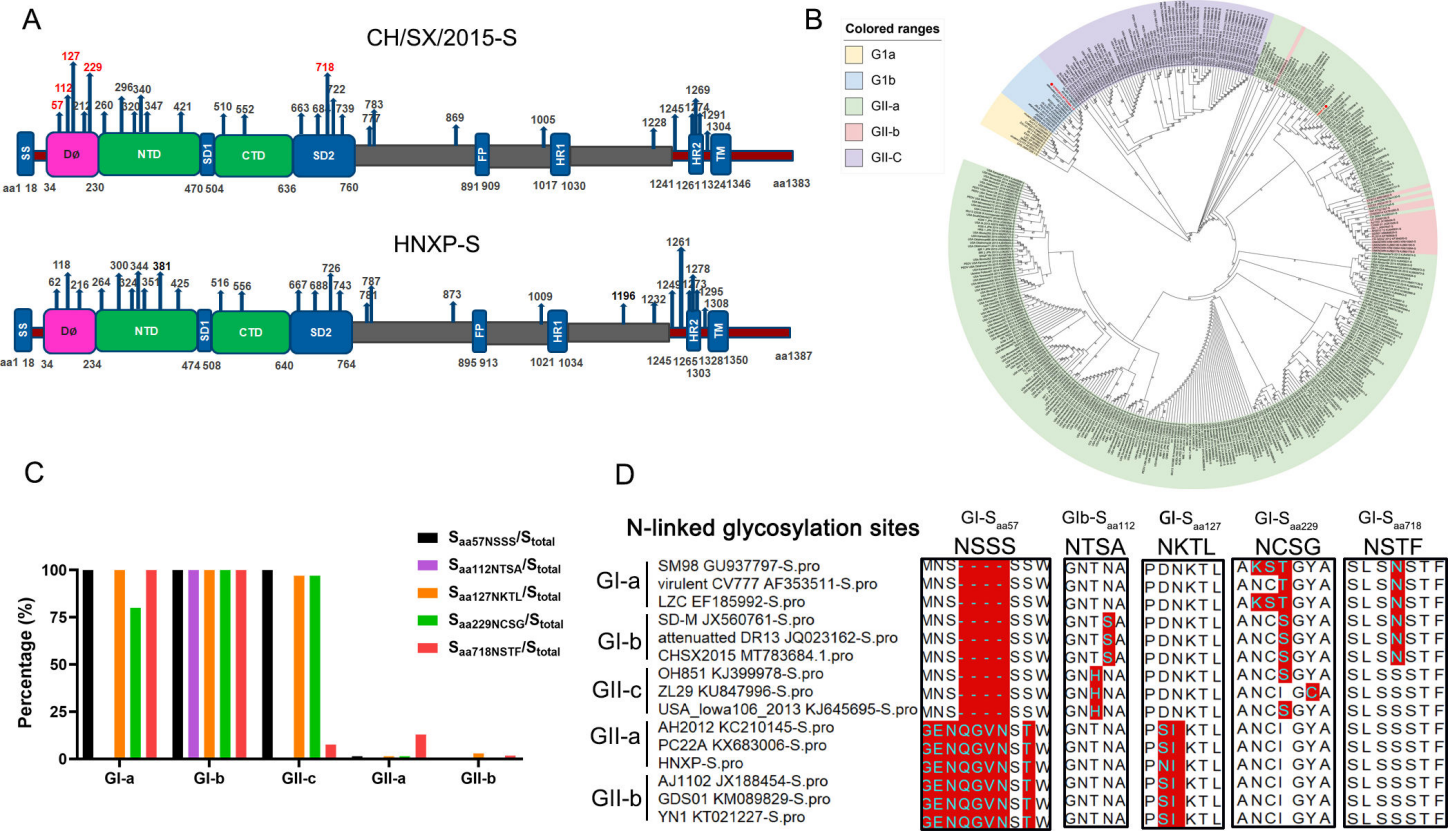
Distribution of mutation sites that appeared in the S protein during the evolution of PEDV from the GI strains to GII strains. (**A**) Domains of the S proteins of the PEDV CH/SX/2015 and rCH/SX/2016-S_HNXP_ (rPEDV-S_wt_) strains; the domain boundaries are indicated below. SS, signal sequence; D0, domain 0; NTD, N-terminal domain of S1; SD1, subdomain 1 of S1; CTD, C-terminal domain of S1; SD2, subdomain 2 of S1; FP, fusion peptide; HR1, heptad repeat 1; HR2, heptad repeat 2; and TM, transmembrane domain. The five red numbers represent the N-linked glycosylation sites unique to the CH/SX/2015 S protein compared to the rPEDV-S_wt_ S protein. (**B**) A neighbor-joining phylogenetic tree was constructed using MEGA X software with 1,000 bootstrap replicates and default parameters based on the amino acid sequences of 395 PEDV S proteins. The red circles indicate the S proteins of the PEDV CH/SX/2015 and rCH/SX/2016-S_HNXP_ (rPEDV-S_wt_) strains in this study. (**C**) Distribution of the five N-linked glycosylation sites among different PEDV subtypes. (**D**) Amino acid sequences of the different subtypes at the five N-linked glycosylation sites.

Our previous research confirmed that the recombinant strain PEDV rCH/SX/2015 is an attenuated strain, whereas rCH/SX/2016-S_HNXP_ is a virulent strain (23). To investigate the molecular mechanism of the attenuation of the rCH/SX/2015 strain, the genome sequences of these two strains were compared. There were many differences between the genomes of the rCH/SX/2015 and rCH/SX/2016-S_HNXP_ strains, including the presence of the confirmed virulence factor E protein (23). Considering that the S protein is glycosylated at many sites, we speculated that one of the reasons for the differences in virulence between the strains rCH/SX/2015 and rCH/SX/2016-S_HNXP_ was a difference in S protein glycosylation. Therefore, the N-linked glycosylation sites of the S proteins of rCH/SX2015 and rCH/SX/2016-S_HNXP_ (rPEDV-S_wt_) strains were predicted using the NetNGlyc 1.0 server. As shown in Fig. 1A, compared to the S protein of rCH/SX2015, the N-linked glycosylation motifs of rPEDV-S_wt_ S protein were predicted to be different at positions aa 62, aa 116, aa 131, aa 233, and aa 722, which correspond to positions aa 57, aa 112, aa 127, aa 229, and aa 718 of the S protein of strain rCH/SX2015. To determine the distribution of these motifs among different subtypes of PEDV, the S proteins of 395 PEDV strains (10 of subtype GI-a, 14 of subtype GI-b, 288 of subtype GII-a, 17 of subtype GII-b, and 66 of subtype GII-c) were compared; among them, CH/SX/2015 belongs to GI-b, and rCH/SX/2016-S_HNXP_ belongs to GII-a (Fig. 1B). The predicted glycosylation of aa 57 in the NSSS motif varied as follows: GI-a strains (100%, 10/10), GI-b strains (100%, 14/14), GII-c strains (100%, 66/66), GII-a strains (1.39%, 4/288), and GII-b strains (0%, 0/17). Compared to these strains, the GII-b and other GII-a strains presented certain differences in amino acid sequence, including a four amino acid insertion (^59^QGVN^62^) and mutation of aa 60 from S to T, which resulted in the ^57^NSSS^60^ motif changing to ^57^ENQGVNST^64^, and both ^57^NSSS^60^ and ^62^NSTW^65^ were predicted to be glycosylation motifs. The predicted N-linked glycosylation motif ^112^NSTA^115^ is present only in GI-b strains (100%, 14/14). The predicted N-linked glycosylation motif ^127^NKTL^130^ was found in all GI-a (100%, 10/10) and GI-b (100%, 14/14) strains and most GII-c (96.97%, 64/66) strains, but in only 4 (1.39%, 4/288) and 5 GII-b strains (2.94%, 5/17). The motif ^229^NCSG^232^ is also present mainly in GI-a (80%, 8/10), GI-b (100%, 14/14), and GII-c (96.97%, 64/66), along with 4 GII-a strains (1.39%, 4/288), whereas this motif was not found in any GII-b strains (0%, 0/17). The proportions of the different PEDV genotypes containing the ^718^NSTF^721^ motif are as follows: GI-a (100%, 10/10), GI-b (100%, 14/14), GII-c (7.58%, 5/66), GII-a (12.85%, 37/288), and GII-b (1.76%, 3/17) (Fig. 1C and D). In summary, these five motifs are located mainly in PEDV GI strains, which were prevalent primarily before 2010, and in GII-c strains that appeared in 2010 (22). Nevertheless, relatively few or no in GII (GII-a and GII-b) strains with high virulence emerged after 2010. The above results indicate that these five motifs may play essential roles in the transformation of PEDV from the GI subtype to the GII subtype.

### Identification of glycosylation sites

**Fig 2:**
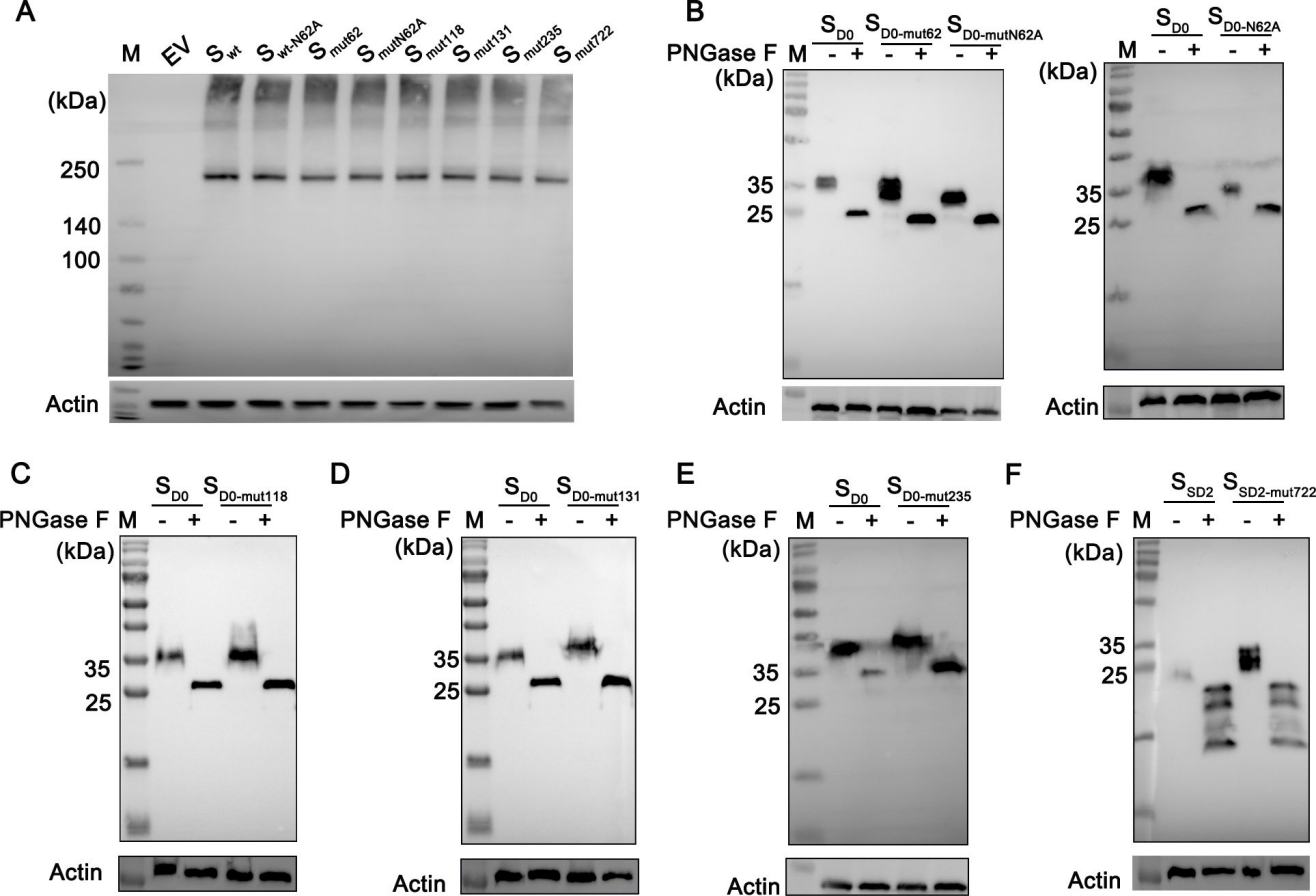
Identification of N-linked glycosylation sites. Plasmids expressing full-length S proteins (pCMV6-EV, pCMV6-S_wt_, pCMV6-S_wt-N62A_, pCMV6-S_mut62_, pCMV6-S_mutN62A_, pCMV6-S_mut118_, pCMV6-S_mut131_, pCMV6-S_mut235_, and pCMV6-S_mut722_) and truncated proteins (pCMV6-S_D0_, pCMV6-S_D0-N62A_, pCMV6-S_D0-mut62_, pCMV6-S_D0-mutN62A_, pCMV6-S_D0-mut118_, pCMV6-S_D0-mut131_, pCMV6-S_D0-mut235_, pCMV6-S_SD2_, and pCMV6-S_SD2-N722S_) were transfected into HEK-293T cells. The mobility of full-length S protein (S_wt_) and mutant proteins (S_wt-N62A_, S_mut62_, S_mutN62A_, S_mut118_, S_mut131_, S_mut235_, and S_mut722_) was analyzed by Western blotting using 7% Tris-acetate gel (**A**). The mobility of the motifs ^57^NSSS^60^ and ^62^NSTW^65^ (**B**), ^116^NTSA^119^ (**C**), ^131^NTSA^134^ (**D**), ^233^NCSG^236^ (**E**), and ^722^NSTF^725^ (**F**) in the S-truncated proteins was analyzed by Western blotting.

The full-length S protein (S_wt_) and mutant proteins (S_wt-N62A_, S_mut62_, S_mutN62A_, S_mut118_, S_mut131_, S_mut235_, and S_mut722_) were transfected into HEK-293T cells, and their expression and mobility were detected via a 7% Tris-acetate gel electrophoresis. The S_wt_ protein and its mutants were all expressed and showed no difference in mobility (Fig. 2A). Because the predicted N-linked glycosylation sites are located in the D0 and SD2 domains (Fig. 1A), respectively, the plasmids expressing the D0 and SD2 domains and their mutants were constructed. Then, the plasmids were transfected into HEK-293T cells, and their expression and mobility were detected. Compared to that of S_D0-mut62_ (^57^ENQGVNST^64^ to ^57^NSSS^60^ modification), the mobility of S_D0-mutN62A_ (^57^ENQGVNST^64^ to ^57^NSSS^60^ modification, then ^57^NSSS^60^ to ^57^ASSS^60^ modification) was changed. Compared to that of S_D0_, the mobility of S_D0-N62A_ (^62^NSTW^65^ to ^62^ASTW^65^ modification) also changed. After treatment with PNGase F, the mobility of the S_D0-mutN62A_ protein was the same as that of the S_D0-mut62_ protein, and the mobility of the S_D0-N62A_ protein was the same as that of the S_D0_ protein, indicating that the differences in mobility are due to the different degrees of glycosylation, thus indicating that the ^57^NSSS^60^ motif in CH/SX/2015 and the ^62^NSTW^65^ motif in rCH/SX/2016-S_HNXP_ were glycosylated (Fig. 2B). The same method was used to further clarify that the mutated motifs ^131^NKTL^134^, ^233^NCSG^236^, and ^722^NSTF^725^ were glycosylated (Fig. 2C through F). The results confirmed that the predicted sites were glycosylated when the proteins were expressed in the forms of the D0 and SD2 domains.

### *In vitro* characterization of the recombinant strains

**Fig 3:**
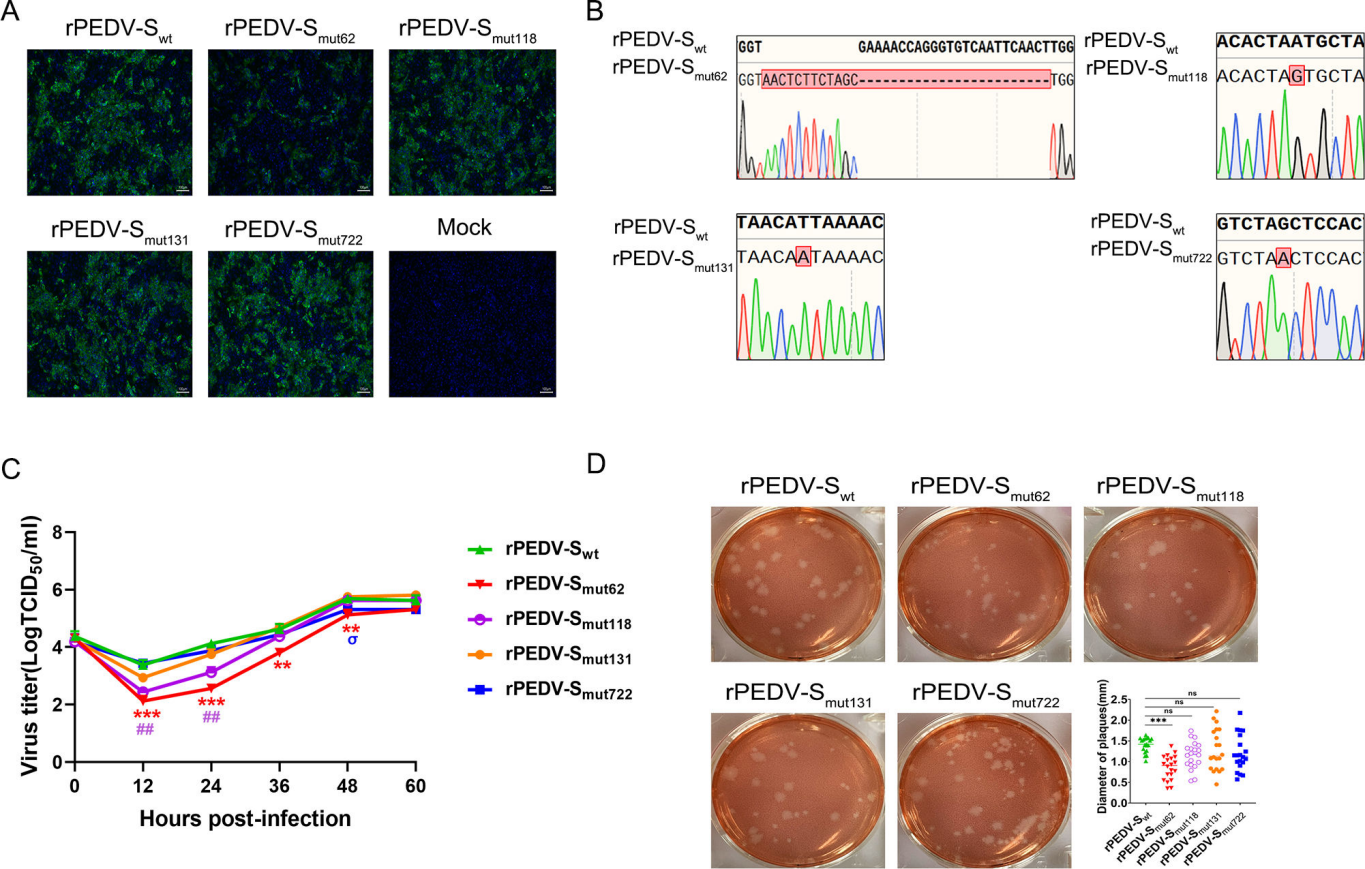
*In vitro* characterization of the recombinant strains. (**A**) VERO cells were infected with rPEDV-S_wt_, rPEDV-S_mut62_, rPEDV-S_mut118_, rPEDV-S_mut131_, and rPEDV-S_mut722_. Infected cells were fixed at 36 hpi and immunolabeled with a fluorescein isothiocyanate (FITC)-AffiniPure goat anti-mouse IgG (H+L). Nuclei were labeled with DAPI (blue). (**B**) VERO cells were infected with rPEDV-S_wt_, rPEDV-S_mut62_, rPEDV-S_mut118_, rPEDV-S_mut131_, and rPEDV-S_mut722_. Infected cells were harvested at 36 hpi. Total RNA was extracted and reverse transcribed. Rescue of the rPEDV-S_mut62_, rPEDV-S_mut118_, rPEDV-S_mut131_, and rPEDV-S_mut722_ strains were confirmed by Sanger sequencing. (**C**) VERO cells in 12-well plates were infected with rPEDV-S_wt_, rPEDV-S_mut62_, rPEDV-S_mut118_, rPEDV-S_mut131_, or rPEDV-S_mut722_ at an MOI of 0.1. The supernatant was harvested at 0, 12, 24, 36, 48, and 60 hpi and titrated with VERO cells. (**D**) Plaques of recombinant PEDVs in VERO cells. The size of the plaque was calculated using ImageJ software. The data were analyzed by one-way ANOVA followed by Dunnett’s multiple comparison test. Each group was compared to the rPEDV-S_wt_ group at the same time point. Significance is indicated as *P < 0.05, **P < 0.01, and ***P < 0.001. The error bars indicate standard deviations.

Considering the changes in the above motifs that occurred during the evolution of virulence of PEDV from the GI strains to GII strains, we speculated that changes in these sites are related to viral virulence. A series of recombinant strains (rPEDV-S_mut62_ [^57^ENQGVNST^64^ to ^57^NSSS^60^ modification], rPEDV-S_mut118_ [^116^NTNA^119^ to ^116^NTSA^119^ modification], rPEDV-S_mut131_ [^131^IKTL^134^ to ^131^NKTL^134^ modification], rPEDV-S_mut235_ [^233^NCIG^236^ to ^233^NCSG^236^ modification], and rPEDV-S_mut722_ [^722^SSTF^725^ to ^722^NSTF^725^ modification]) were constructed using the rPEDV-S_wt_ strain as the backbone. Immunofluorescence assay (IFA) (Fig. 3A) and Sanger sequencing (Fig. 3B) revealed that four recombinant strains were successfully rescued, with the exception of rPEDV-S_mut235_. To understand the growth properties of these PEDV recombinant strains in VERO cells, VERO cells were infected with rPEDV-S_wt_ or the recombinant strains at a multiplicity of infection (MOI) of 0.1. At 0, 12, 24, 36, 48, and 60 h post-infection (hpi), the cell supernatants were collected, and the viral titer in the cell supernatants was measured to monitor the multistep growth kinetics. The results revealed no difference in virus titer among all the groups at 0 hpi, indicating that the titer of each virus was similar at the initial infection. Compared to that of rPEDV-Swt, the titer of rPEDV-S_mut62_ decreased at 12, 24, 36, and 48 hpi, the titer of rPEDV-S_mut118_ decreased at 12 and 24 hpi, and the titer of rPEDV-S_mut722_ decreased at 48 hpi (Fig. 3C). The plaque phenotypes of these recombinant strains were further explored. Compared to rPEDV-S_wt_, only rPEDV-S_mut62_ formed smaller plaques (Fig. 3D). In brief, these results demonstrated that rPEDV-S_mut62_, rPEDV-S_mut118_, rPEDV-S_mut131_, and rPEDV-S_mut722_ were successfully rescued.

### The effects of the recombinant strains were attenuated in piglets

**Fig 4:**
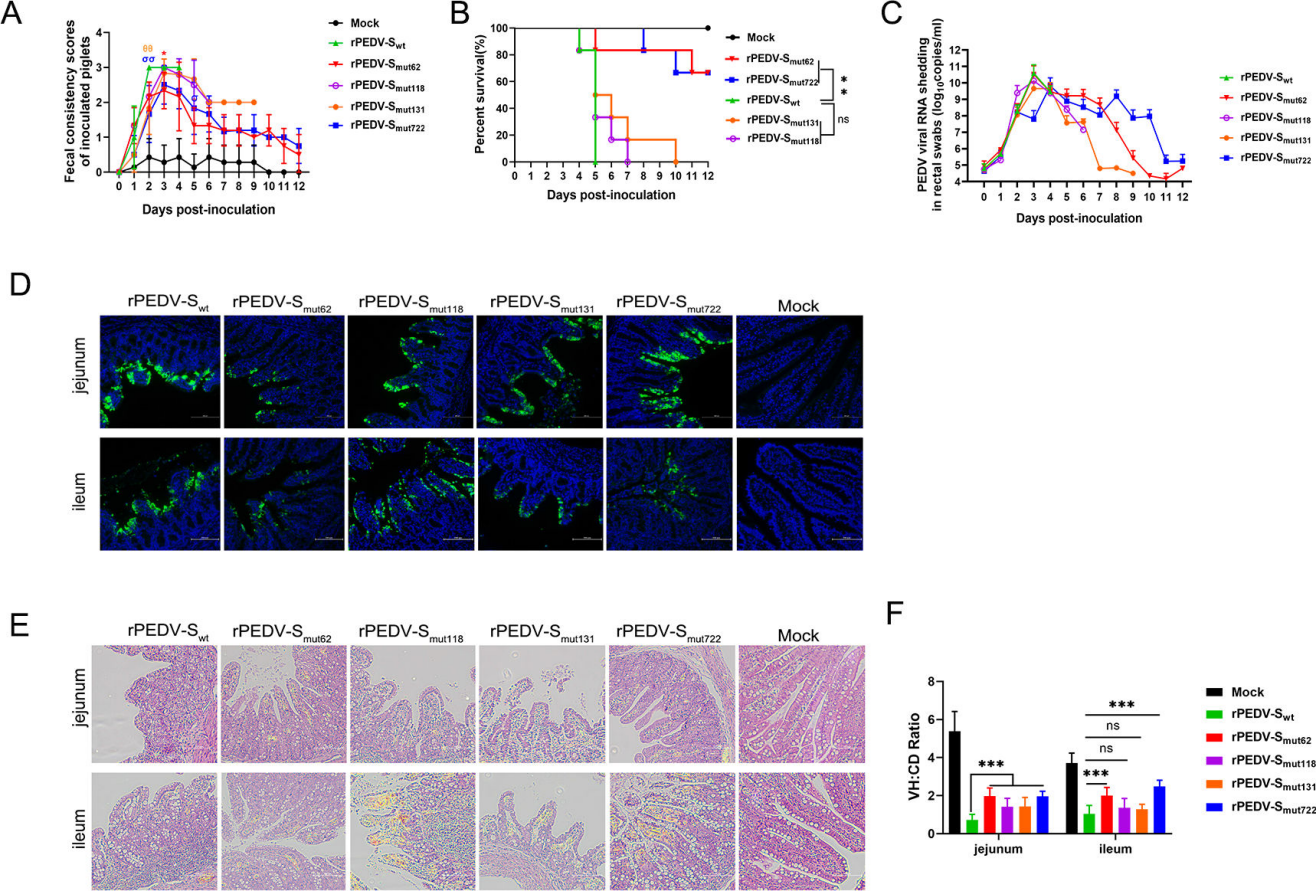
Pathogenicity of the recombinant strains in piglets. (**A**) Fecal scores of the pigs. The fecal scores were determined as follows: 0, solid; 1, pasty; 2, semiliquid; and 3, liquid. Each line indicates the mean score of a group. Asterisks (*) indicate significant differences between rPEDV-S_wt_ and rPEDV-S_mut62_ (*P < 0.05). Thetas (*θ*) indicate significant differences between rPEDV-S_wt_ and rPEDV-S_mut131_ (^θθ^*P* < 0.01). Sigmas (*σ*) indicate significant differences between rPEDV-S_wt_ and rPEDV-S_mut722_ (^σσ^*P* < 0.01). (**B**) Survival curves of the piglets. The data were analyzed using the log-rank test (**P < 0.01). (**C**) Fecal shedding of PEDV RNA. Viral RNA was isolated from rectal swab samples daily and subjected to RT‒qPCR to determine the number of PEDV N gene RNA copies. (**D**) Immunohistochemical staining images of the PEDV N protein in the jejunum and ileum. The number of antigen signals was calculated using ImageJ software. (**E**) H&E staining of the jejunum and ileum from dying or euthanized piglets. (**F**) VH:CD ratios of the piglets. Ten villi from each intestinal section were measured. The differences among groups were analyzed by one-way ANOVA followed by Dunnett’s multiple comparison test. Each group was compared to the rPEDV-S_wt_ group at the same time point. The error bars indicate standard deviations.

To assess the pathogenicity of these recombinant strains, 37 newborn piglets were randomly divided into six groups, with 7 pigs in the mock group and 6 pigs in the other groups. The piglets were orally inoculated with rPEDV-S_wt_ or a recombinant strain at a dose of 10^4^ TCID_50_, while the piglets in the mock group were given an equal volume of DMEM. All the piglets in the rPEDV-S_wt_ group developed severe diarrhea (100% [6/6]) and died (100% [6/6]) within 5 dpi (Fig. 4A and B)**.** Although the final diarrhea rates in all the recombinant strain groups were 100%, diarrhea peaked on the third day after infection, whereas all the piglets in the rPEDV-S_wt_ group experienced watery diarrhea on the second day. Compared to the rPEDV-S_wt_ group (average diarrhea score = 3), the other groups presented relatively mild diarrhea on the second day: rPEDV-S_mut62_ (average diarrhea score = 2.167), rPEDV-S_mut118_ (average diarrhea score = 2.167), rPEDV-S_mut131_ (average diarrhea score = 1.833), and rPEDV-S_mut722_ (average diarrhea score = 1.667). Furthermore, compared to the rPEDV-S_wt_ group (average diarrhea score = 3), the rPEDV-S_mut62_ (average diarrhea score = 2.333) and rPEDV-S_mut722_ (average diarrhea score = 2.5) presented relatively mild diarrhea on the third day (Fig. 4A). In addition, only two pigs (33% [2/6]) in the rPEDV-S_mut62_ inoculated group died, one each on days 5 and 11. The piglets in the rPEDV-S_mut118_ (100% [6/6]) and rPEDV-S _mut131_(100% [6/6]) groups all died on the 7th and 10th days after infection. Two piglets (33% [2/6]) in the rPEDV-S_mut722_ died, one each on days 8 and 10 (Fig. 4B). With the exception of the rPEDV-S_mut722_ group, in which viral shedding peaked on the fourth day after infection, viral RNA fecal shedding peaked on the third day after infection in the other groups. In addition, compared to the highest level of viral shedding, which occurred in the rPEDV-S_wt_ group (4.7 × 10^10^ copies/mL), the rPEDV-S_mut62_ group (3.2 × 10^10^ copies/mL), the rPEDV-S_mut118_ group (1.4 × 10^10^ copies/mL), the rPEDV-S_mut131_ group (4.6 × 10^9^ copies/mL), and the rPEDV-S_mut722_ group (6.5 × 10^9^ copies/mL) exhibited lower viral RNA fecal shedding (Fig. 4C). Immunohistochemistry (IHC) staining revealed that all of the infected epithelial cells exhibited wide distribution of the PEDV antigen. The average optical density (AOD) was calculated to indicate positive antigen staining in the small intestine. The AODs in the jejunums of the rPEDV-S_mut62_ (10.573), rPEDV-S_mut118_ (14.215), and rPEDV-S_mut131_ (12.543) groups were lower than that of the rPEDV-S_wt_ group (20.855). Furthermore, the AODs in the ileums of the rPEDV-S_mut62_ (10,764), rPEDV-S_mut131_ (7.338), and rPEDV-S_mut722_ (12.755) groups were lower than that of the rPEDV-S_wt_ group (14.816) (Fig. 4D). Hematoxylin and eosin (H&E) staining revealed that all four recombinant strains caused milder histopathological lesions to the intestinal villi in the jejunum, but only the rPEDV-S_mut62_ and rPEDV-S_mut722_ strains caused milder histopathological lesions to the intestinal villi than did the rPEDV-S_wt_ strain in the ileum (Fig. 4E). The villous height/crypt depth (VH/CD) ratio in the jejunum of the rPEDV-S_wt_ group was markedly lower than those in the other infection groups. The VH/CD ratio in the ileum of the rPEDV-S_wt_ group was significantly lower than those in the rPEDV-S_mut62_ and rPEDV-S_mut722_ groups but did not differ from those in the rPEDV-S_mut118_ and rPEDV-S_mut131_ groups (Fig. 4F). Collectively, these data suggested that the virulence of the recombinant strains rPEDV-S_mut62_ and rPEDV-S_mut722_ was mildly attenuated in neonatal piglets.

### The rPEDV-S_mut62_ and rPEDV-S_mut722_ strains protected pigs against wild-type virus challenge

**Fig 5:**
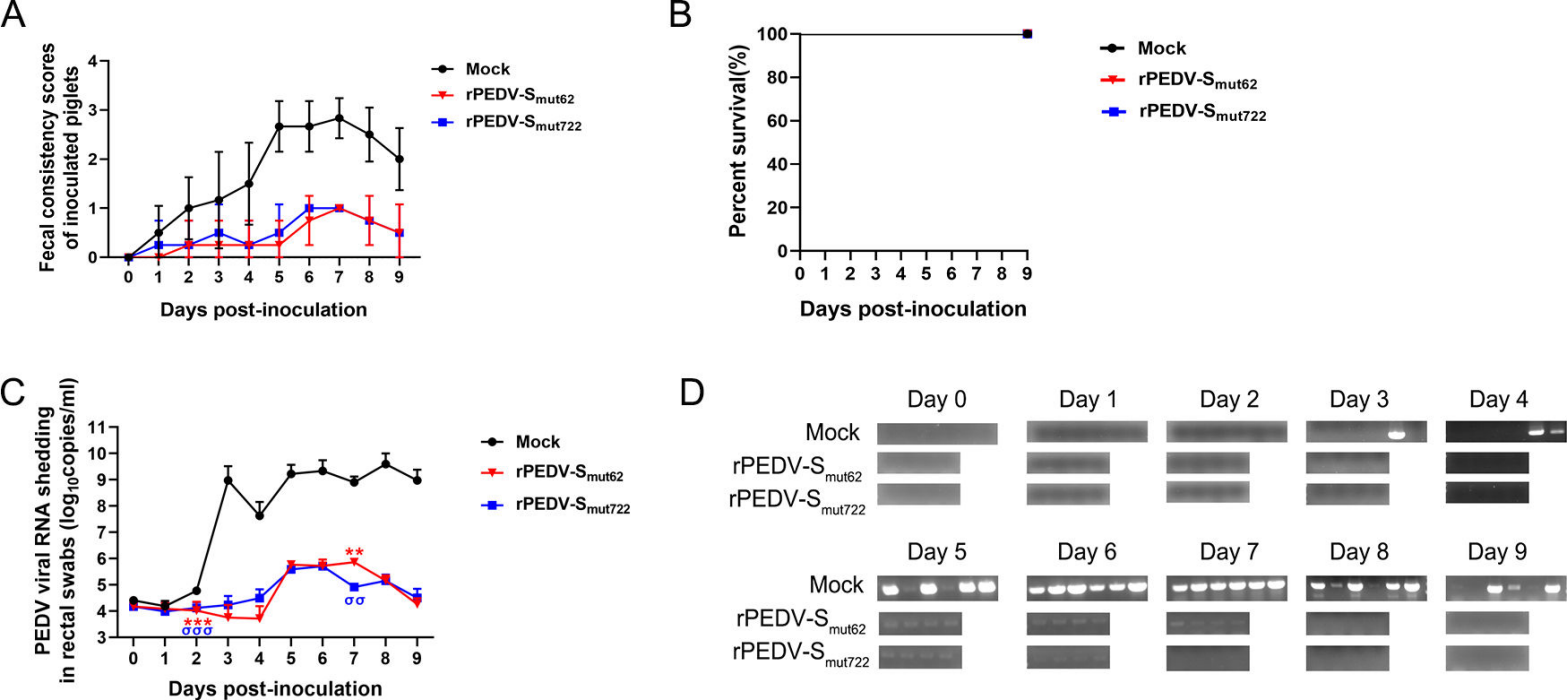
Clinical signs of piglets after rPEDV-S_wt_ challenge. (**A**) Fecal scores of piglets on different days post-challenge (dpc). Fecal scores were scored as follows: 0, solid; 1, pasty; 2, semiliquid; and 3, liquid. Each line indicates the mean score of a group. (**B**) Survival curves of piglets on different dpc. (**C**) and (**D**) Evaluation of PEDV RNA shedding in pig feces post-challenge by RT–qPCR and RT‒PCR. The differences among groups were analyzed using an unpaired *t* test. Asterisks (*) indicate significant differences between mock and rPEDV-S_mut62_. Sigmas (σ) indicate significant differences between mock and rPEDV-S_mut722_. Significance is indicated as ^*/σ^*P* < 0.05, ^**/σσ^*P* < 0.01, and ^***/σσσ^*P* < 0.001. The error bars indicate standard deviations.

**Fig 6:**
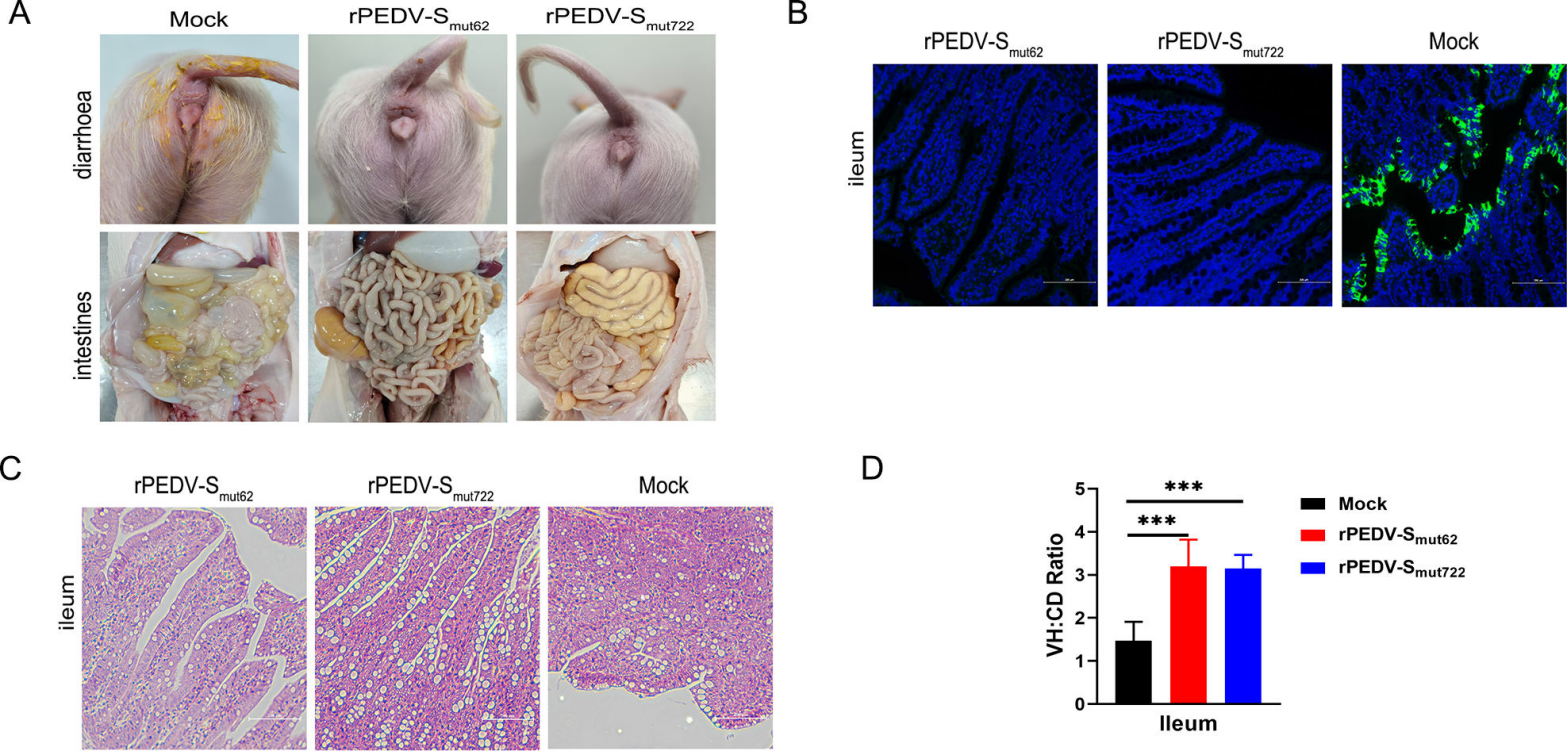
Changes in piglet intestines 9 days after rPEDV-S_wt_ challenge. (**A**) Clinical diarrhea and gross lesions of the small intestine. (**B**) Immunohistochemical staining images of the PEDV N protein in the ileum. (**C**) H&E staining images of the ileums from dying or euthanized piglets. (**D**) VH:CD ratios of the piglets. Ten villi from each intestinal section were measured. The differences among groups were analyzed by one-way ANOVA followed by Dunnett’s multiple comparison test. Each group was compared to the rPEDV-S_wt_ group at the same time point. The significance is indicated as *P < 0.05, **P < 0.01, and ***P < 0.001. The error bars indicate standard deviations.

At 21 dpi, the remaining pigs were challenged with the virulent rPEDV-S_wt_ strain at the high dose of 1 × 10^6^ TCID_50_/pig. One pig in the mock-challenged group began to experience diarrhea at 3 days post-challenge (dpc), and all pigs in the mock-challenged group experienced severe diarrhea (average diarrhea score >2) at 5–9 dpc (100% [6/6]), which improved at 9 dpc. Moreover, no pigs experienced diarrhea (average diarrhea score <1) in the rPEDV-S_mut62_ and rPEDV-S_mut722_ groups (Fig. 5A). All of the piglets in each group did not die at the end of the study (Fig. 5B). Moreover, compared to the highest level of viral shedding (4.0 × 10^9^ copies/mL), which occurred in the mock-challenged group, the pigs in the rPEDV-S_mut62_ (7.4 × 10^5^ copies/mL) and rPEDV-S_mut722_ (5.1 × 10^5^ copies/mL) groups presented significantly less PEDV RNA shedding (Fig. 5C and D). There was residual diarrhea feces on the anus of the mock-challenged piglets, whereas the anuses of the piglets in the rPEDV-S_mut62_ and rPEDV-S_mut722_ groups were clean (Fig. 6A). At 9 dpc, all the pigs were sacrificed and necropsied, and the rPEDV-S_mut62_ and rPEDV-S_mut722_ groups of pigs presented no significant gross lesions in their intestinal tissue, whereas thin-walled and gas-distended intestines were observed in the piglets from the mock-challenged group (Fig. 6A). IHC staining showed the wide distribution of the PEDV antigen in the ileum epithelial cells in the mock-challenged group. However, no antigens were detected in the ileum epithelial cells of pigs in either the rPEDV-S_mut62_ or the rPEDV-S_mut722_ groups (Fig. 6B). H&E staining revealed that there was almost no damage to the intestinal villi of the pigs in either the rPEDV-S_mut62_ or the rPEDV-S_mut722_ groups, whereas the pigs in the mock-challenged group presented more severe damage (Fig. 6C). The VH/CD ratio in the ileum of the mock-challenged group was significantly lower than those of the rPEDV-S_mut62_ and rPEDV-S_mut722_ groups (Fig. 6D). In general, the rPEDV-S_mut62_ and rPEDV-S_mut722_ strains protected against wild-type virus challenge in pigs.

### The rPEDV-S_mut62_ and rPEDV-S_mut722_ strains induce high levels of SlgA, IgG, and effective cross-protection neutralizing antibodies

**Fig 7:**
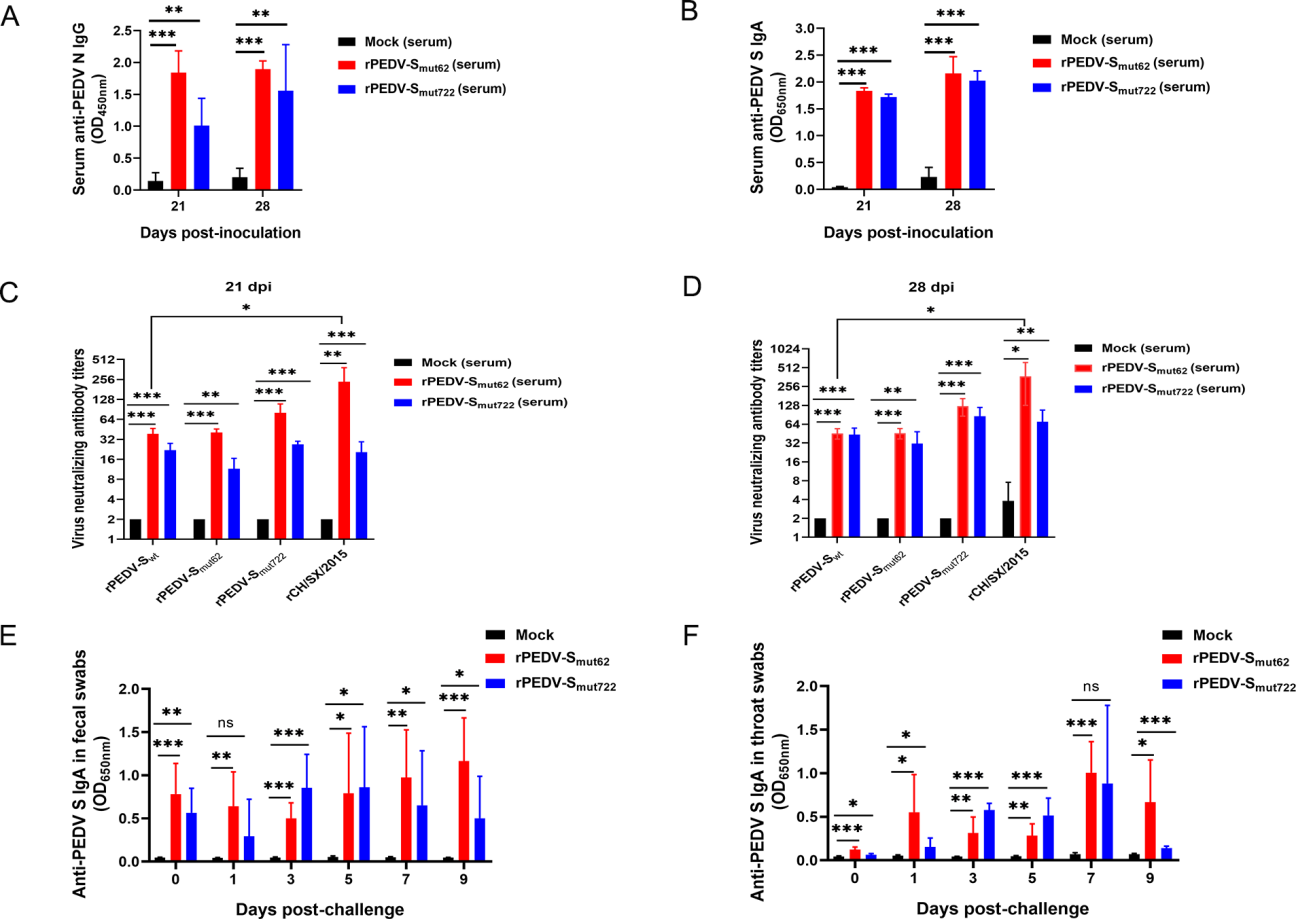
Protection induced by the rPEDV-S_mut62_ and rPEDV-S_mut722_ strain against rPEDV-S_wt_ challenge in pigs. (**A** and **B**) The levels of anti-PEDV N IgG and PEDV S IgA in sera collected at 21 and 28 dpi. (**C** and **D**) Viral neutralizing antibody titer against the PEDV GI and GII strains in serum collected at 21 and 28 dpi (7 dpc). (**E** and **F**) Levels of SlgA secreted in feces and saliva. The data were analyzed by one-way ANOVA followed by an unpaired *t* test. Significance is indicated as *P < 0.05, **P < 0.01, and ***P < 0.001. The error bars indicate standard deviations.

To determine whether piglets infected with the rPEDV-S_mut62_ and rPEDV-S_mut722_ exhibit an antibody response, serum samples were collected at 21 dpi/0 dpc and 28 dpi/7 dpc, and then the titer of anti-PEDV N IgG, anti-PEDV S IgA, and viral neutralization antibodies in the serum were determined. The piglets in the rPEDV-S_mut62_ and rPEDV-S_mut722_ groups presented significantly higher serum levels of anti-PEDV N IgG and S IgA than those in the mock group at 21 dpi/0 dpc and 28 dpi/7 dpc (Fig. 7A and B). The serum of the rPEDV-S_mut62_ and rPEDV-S_mut722_ groups elicited robust neutralizing antibody responses to the rPEDV-S_wt_, rPEDV-S_mut62_, rPEDV-S_mut722_, and CH/SX/2015 strains. Moreover, the ability of rPEDV-S_mut62_ serum to neutralize the CH/SX/2015 strain was higher than that of the rPEDV-S_wt_ strain at 21 dpi/0 dpc and 28 dpi/7 dpc (Fig. 7C and D). Secretory immunoglobulin A (SlgA) is crucial for protecting against PEDV. Therefore, throat and fecal swabs were collected, and the SlgA levels in saliva and feces were detected. Compared to the mock-challenged group, the rPEDV-S_mut62_ and rPEDV-S_mut722_ groups presented significantly higher levels of SlgA in saliva and feces. Moreover, the level of SlgA increased after challenging the virulent rPEDV-S_wt_ strain (Fig. 7E and F). In summary, the rPEDV-S_mut62_ and rPEDV-S_mut722_ strains induced cross-protection against the GI and GII subtypes of PEDV.

### The recombinant strains were genetically stable after serial passaging *in vitro* and *in vivo*

To evaluate the genetic stability of the recombinant strains rPEDV-S_wt_, rPEDV-S_mut62_, rPEDV-S_mut118_, rPEDV-S_mut131_, and rPEDV-S_mut722_ *in vitro*, the recombinant strains were serially passaged from P1 to P10 in VERO cells. The whole-genome sequences of the recombinant strains from P5 to P10 were determined via Sanger sequencing, and the results revealed no reversion mutations at positions aa 62, aa 118, aa 131, and aa 722 of the S proteins of these strains (Table 1). However, one mutation (G965S) was identified in the S protein of rPEDV-S_mut62_, and one mutation (S70L) was identified in the E protein of the rPEDV-S_mut722_ strain at P10. No mutations were detected in the other three strains (Table 2). Moreover, the genetic stability of the mutants was evaluated by sequencing rectal swab samples collected from piglets infected with the recombinant strains at the indicated times: rPEDV-S_mut62_ (8 dpi), rPEDV-S_mut118_ (6 dpi), rPEDV-S_mut131_ (8 dpi), and rPEDV-S_mut722_ (8 dpi). The results revealed that there were no reversion mutations in the corresponding mutation motifs of the recombinant strains (Table 1). However, two mutations K1028N and D1172A were identified in the S protein from the rectal swab samples of rPEDV-S_mut62_ and rPEDV-S_mut722_ infected piglets at 8 dpi, respectively (Table 2). Collectively, the results from passaging the recombinant strains in either cell culture or piglets revealed that the recombinant strains were genetically stable.

**TABLE 1:** Sequencing of the reversions in the recombinant viruses^*a*^

Nucleotide location (S)	Wild type	Mutant type	Passages/days
rPEDV-S_mut62_ (bp 163–183)/(aa 54–61)	ACTGGT**GAAAACCAGGGTGTCAATTCAACT**TGG (TG**ENQGVNST**W)	ACTGGT**AACTCTTCTAGC**TGG (TG**NSSS**W)	P5
rPEDV-S_mut62_ (bp 163–183)/(aa 54–61)	ACTGGT**GAAAACCAGGGTGTCAATTCAACT**TGG (TG**ENQGVNST**W)	ACTGGT**AACTCTTCTAGC**TGG (TG**NSSS**W)	P10
rPEDV-S_mut118_ (bp 346–363)/(aa 116–122)	AACGGTAACACT**AAT**GCT (NGNT**N**A)	AACGGTAACACT**A****ST**GCT (NGNT**S**A)	P5
rPEDV-S_mut118_ (bp 346–363)/(aa 116–122)	AACGGTAACACT**AAT**GCT (NGNT**N**A)	AACGGTAACACT**A****ST**GCT (NGNT**S**A)	P10
rPEDV-S_mut131_ (bp 385–402)/(aa 129–134)	CCTAAC**ATT**AAAACATTG (PN**I**KTL)	CCTAAC**AAT**AAAACATTG (PN**N**KTL)	P5
rPEDV-S_mut131_ (bp 385–402)/(aa 129–134)	CCTAAC**ATT**AAAACATTG (PN**I**KTL)	CCTAAC**AAT**AAAACATTG (PN**N**KTL)	P10
rPEDV-S_mut722_ (bp 2158–2175)/(aa 719–725)	TTGTCT**AGC**TCCACTTTT (LS**S**STF)	TTGTCT**AAC**TCCACTTTT (LS**N**STF)	P5
rPEDV-S_mut722_ (bp 2158–2175)/(aa 719–725)	TTGTCT**AGC**TCCACTTTT (LS**S**STF)	TTGTCT**AAC**TCCACTTTT (LS**N**STF)	P10
rPEDV-S_mut62_ (bp 163–183)/(aa 54–61)	ACTGGT**GAAAACCAGGGTGTCAATTCAACT**TGG (TG**ENQGVNST**W)	ACTGGT**AACTCTTCTAGC**TGG (TG**NSSS**W)	D8
rPEDV-S_mut118_ (bp 346–363)/(aa 116–122)	AACGGTAACACT**AAT**GCT (NGNT**N**A)	AACGGTAACACT**A****ST**GCT (NGNT**S**A)	D6
rPEDV-S_mut131_ (bp 385–402)/(aa 129–134)	CCTAAC**ATT**AAAACATTG (PN**I**KTL)	CCTAAC**AAT**AAAACATTG (PN**N**KTL)	D6
rPEDV-S_mut722_ (bp 2158–2175)/(aa 719–725)	TTGTCT**AGC**TCCACTTTT (LS**S**STF)	TTGTCT**AAC**TCCACTTTT (LS**N**STF)	D8

^*a*^ The mutations are marked in bold and underlined.

**TABLE 2:** Mutations in the remaining genomes of the recombinant viruses of P10 or day 8

Nucleotide location (S)	Wild type	Mutant type	Passages/days
rPEDV-S_mut62_ (bp 2887–2898)/(aa 963–966)	CTAGGA**GGT**TTT (LG**G**F)	CTAGGA**AGT**TTT (LG**S**F)	P10
rPEDV-S_mut722_ (E) (bp 25649–25667)	CCCCTCCCTAGTACTGTT (PLP**S**TV)	CCCCTCCTTAGTACTGTT (PLP**L**TV)	P10
rPEDV-S_mut62_ (bp 3076–3090)/(aa 1026–1034)	ACTTCCAAGGGTTTG (TS**K**GL)	ACTTCCAATGGTTTG (TS**N**GL)	D8
rPEDV-S_mut722_ (bp 3508–3522)/(aa 1170–1174)	GCCATCGATGGCTTA (AI**D**GL)	GCCATCGCTGGCTTA (AI**A**GL)	D8

^*a*^ The mutations are marked in bold and underlined.

### The change in the ^57^ENQGVNST^64^ motif to ^57^NSSS^60^ and from the ^718^SSTF^721^ motif to ^722^NSTF^725 ^alters the homotrimeric structure of the S protein

**Fig 8:**
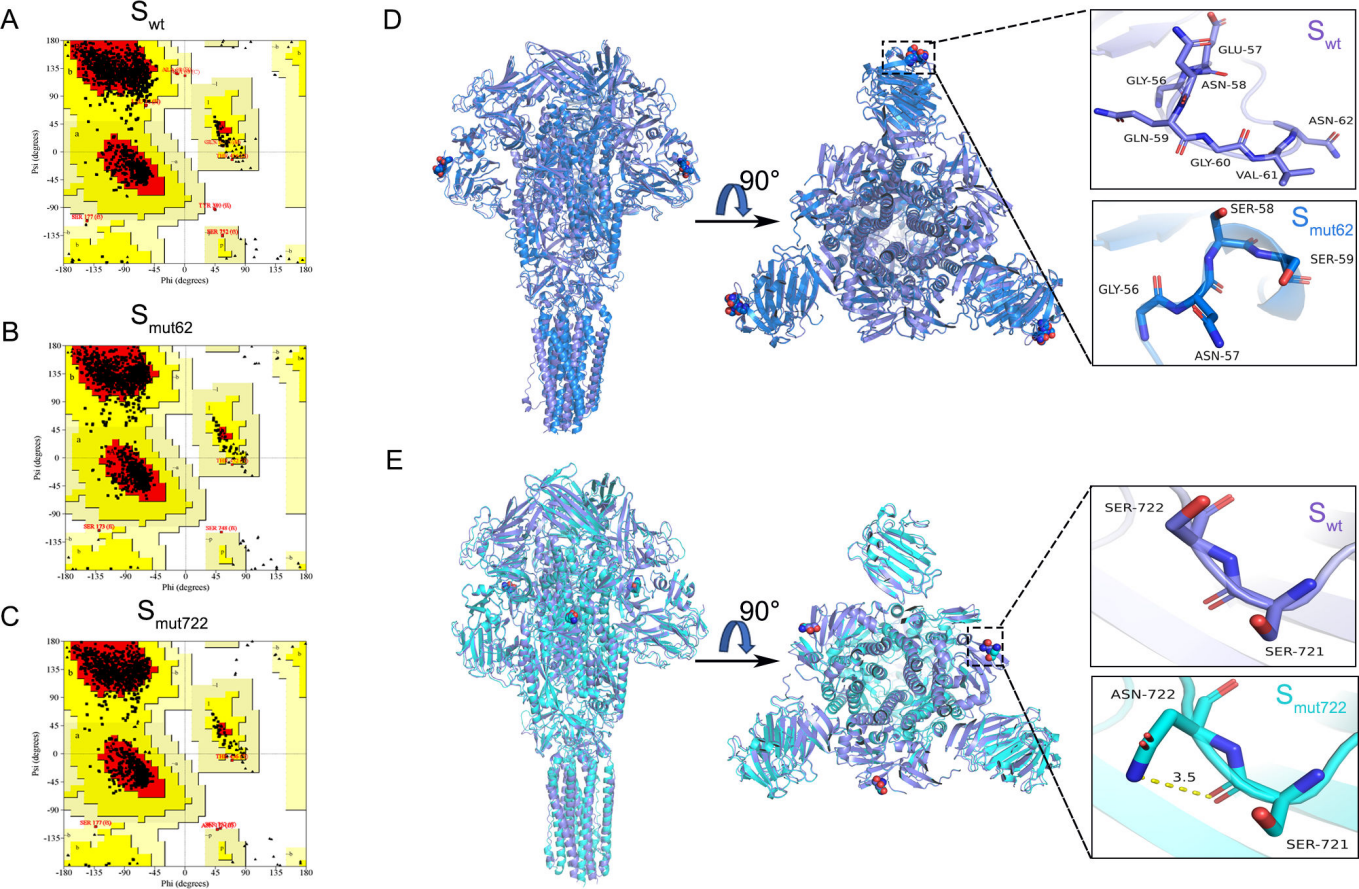
Structural prediction of the PEDV S_wt_, S_mut62_, and S_mut722_ proteins using AlphaFold 3. (**A–C**) Ramachandran plots of the predicted structures of PEDV S_wt_, S_mut62_, and S_mut722_. The red, yellow, light yellow, and white regions represent the favored, allowed, “generously allowed,” and unallowed regions, respectively. (**D**) Overlay of the predicted structures of S_wt_ and S_mut62_ and an enlarged view of aa 57–62. (**E**) Overlay of the predicted structures of S_wt_ and S_mut722_ and an enlarged view of aa 721–722.

To further analyze the molecular mechanism of rPEDV-S_mut62_ and rPEDV-S_mut722_ attenuated virulence, the trimeric S proteins of the recombinant viruses were constructed using AlphaFold 3. Ramachandran plot results revealed that approximately 99.9% of the amino acid residues in S_wt_, S_mut62_, and S_mut722_ were in the reasonable region, indicating that the predicted protein structures are highly reliable and can serve as a template for research (Fig. 8A through C). The PyMOL was used to superimpose the structures of the S_wt_ and S_mut62_ homotrimeric proteins, and the results revealed that the overall root mean square deviation (RMSD) between them was 0.385, suggesting good overlap. The aa 57–59 segment of the S_wt_ protein adopted an α-helical structure, whereas the corresponding position of the S_mut62_ protein adopted a loop structure, which indicates that the structure at this location had undergone significant changes (Fig. 8D). The same method was used for comparing the S_wt_ and S_mut722_ proteins, and the RMSD was 0.337, suggesting good overlap between S_wt_ and S_mut722_. Although the trimeric structures of these two proteins remained unchanged near aa 722, there were significant changes to the hydrogen bonding interactions. aa 722 of the S_wt_ protein is a polar uncharged serine residue that does not form any polar interactions with the surrounding amino acids. However, aa 722 of S_mut722_ is a polar uncharged asparagine residue, and the amino group of the asparagine side chain forms a hydrogen bond with serine at position 721, with a distance of 3.5 Å (Fig. 8E). Therefore, these structural changes may lead to changes in the performance of rPEDV-S_mut62_ and rPEDV-S_mut722_.

## DISCUSSION

PEDV remains a significant concern that affects the development of healthy animals in the pork industry worldwide. Immunization with safe and effective vaccines is the main approach for controlling the spread of PEDV. However, the available vaccines cannot provide complete protection against newly emerging virulent strains of PEDV. The main reason for this is the high degree of variation in the S protein. Before 2010, PED caused by the GI genogroup was only sporadic outbreaks and not large-scale epidemics. Since 2010, GII PEDV variants have spread worldwide and caused significant economic losses. The PEDV S protein is a glycoprotein and a major virulence factor (6, 25). Therefore, we predicted the glycosylation sites of the S protein. Compared to the S proteins of the GI strains, five predicted N-linked glycosylation motifs changed in the PEDV GII strains. We further confirmed that the motifs ^57^NSSS^60^, ^131^NKTL^134^, ^233^NCSG^236^, and ^722^NSTF^725^ were glycosylated by expressing the D0 and SD2 domains (Fig. 2). When expressed by themselves, these domains may not fold properly compared to the full-length protein. We also expressed the full-length S protein and its mutants, but there was almost no difference in mobility (Fig. 2A). The possible reason is that the S protein is highly glycosylated, and changes in the mobility of the full-length S protein caused by modification of a single site may not be observable. Due to the four amino acid insertion ^59^QGVN^62^ mutation of aa 60 from S to T in the GII PEDV S protein, the motif at this site significantly changes. However, both the GI and GII strains have glycosylation sites here (^57^NSSS^60^ and ^62^NSTW^65^, respectively), but we cannot confirm how these differences in their motifs affect the virulence of PEDV.

The PEDV S1 subunit comprises domain 0 (D0; residues aa 34–233), the N-terminal domain, and the C-terminal domain with subdomains SD1 and SD2 (25). The D0 domain is responsible for sialic acid binding and has been confirmed to be a potential virulence determinant of PEDV strains (10, 26). Deletion of the D0 domain reduces the replication efficiency of PEDV and results in the loss of critical epitopes that induce protective immune responses (9, 27). The region containing aa 56–62 located in the D0 domain is highly variable and may be a key site driving the variations among PEDV strains. Compared to rPEDV-S_wt_, rPEDV-S_mut62_ has a reduced ability to replicate (Fig. 3), possibly due to a mutation at this site that alters the trimeric structure of the S protein (Fig. 8B), thereby affecting its binding to sialic acid. Notably, the virulence of rPEDV-S_mut62_ is attenuated, but it maintains good immunogenicity and can provide piglets with complete protection against virulent rPEDV-S_wt_ challenge (Fig. 4 to 6). Because rPEDV-S_mut62_ contains the ^59^QGVN^62^ insertion and aa 60 S to T mutation, it is impossible to determine which change caused the attenuation of rPEDV-S_mut62_ virulence; thus, further research is needed. In addition, six neutralizing epitopes of the S protein have been identified, including D0 (aa 19–220), S1^A^ (aa 435–485), the COE domain (aa 499–638), the epitope SS2 (aa 748–755), the epitope SS6 (aa 764–771), and the epitope 2C10 (aa 1368–1374) (28, 29). Changing the glycosylation sites in the RBD of the CoV spike protein can affect the production of IgG and neutralization titers *in vivo* (18, 30). The neutralizing antibodies produced by piglets infected with rPEDV-S_mut62_ effectively neutralized PEDV strains of both the GI and GII subtypes (Fig. 7C and D), indicating that the ^57^NSSS^60^ motif may be one of the important regions for neutralizing the epitope D0.

The rPEDV-S_mut722_ strain showed attenuated virulence in piglets and could completely protect piglets against rPEDV-S_wt_ challenge. The corresponding mutated motif ^722^NSTF^726^ is located in the SD2 subdomain (Fig. 1). The SD2 subdomain participates in hinge-like motions that promote the conversion of the SARS-CoV-2 RBD between closed and opened conformations (31). Mutation to aa 722 affects the interactions with surrounding amino acids (Fig. 8C), which may affect the conformation of the RBD (open or closed) and lead to changes in virulence. Little is known about the function of PEDV SD2, but our research has established a clear correlation between SD2 and PEDV virulence.

Many viruses enter the host (human or animal) body through mucosal surfaces (32). The fecal-oral route is the main route of PEDV infection. SlgA is essential for defending against viruses that infect mucosal surfaces. The rPEDV-S_mut62_ and rPEDV-S_mut722_ strains induced high levels of SIgA in saliva, and SIgA plays a crucial role in the first step of PEDV infection. In addition, SIgA antibodies enter the colostrum via the gut-mammary-SIgA axis and provide lactogenic immunity to suckling piglets (33, 34). Compared to those in the mock-challenged group, the piglets in the rPEDV-S_mut62_ and rPEDV-S_mut722_ groups presented high levels of both IgA in the serum and SlgA in the throat and fecal swabs (Fig. 7). These findings indicate that rPEDV-S_mut62_ and rPEDV-S_mut722_ trigger a systemic mucosal immune response. We can infer that the rPEDV-S_mut62_ and rPEDV-S_mut722_ strains may also induce SIgA production in the breast, which indicates that these two strains are promising live attenuated vaccine candidates. The nsp1, nsp2, nsp14, nsp15, nsp16, S, and E genes of PEDV are closely related to viral virulence (35–37). It has been confirmed that the NTD and endocytosis signal of the S protein affect viral virulence (8, 9), and this study further clarifies that the D0 and SD2 domains in the S protein also affect virus virulence. However, in addition to those mentioned above, there are still many unexplored virulence factors of PEDV. Owing to their ability to induce broad and prolonged protective immunity, live attenuated vaccines are promising for controlling PEDV infections. However, safety is the main issue. A highly attenuated virus, which does not cause diarrhea in animals and elicits a neutralizing antibody response in virus-infected animals, may be produced by altering multiple virulence factors via a reverse genetic system.

In summary, this study revealed that the recombinant strains rPEDV-S_mut62_ and rPEDV-S_mut722_ were mildly attenuated and induced cross-protection in piglets. These findings suggest that the motifs ^57^ENQGVNST^64^ and ^722^SSTF^725^ of the S protein in GII strains play roles in the virulence evolution of PEDV and can serve as important targets for the design of live attenuated PEDV vaccines.

## MATERIALS AND METHODS

### Cells and viruses

VERO cells were preserved in our laboratory and cultured in Dulbecco’s modified Eagle medium supplemented with 10% fetal bovine serum, 100 U/mL penicillin, and 100 µg/mL streptomycin. PEDV strain CH/SX/2015 (GenBank no. MT783684) and CH/SX/2016 strain (GenBank no. MT787025) were stored in our laboratory. PEDV HNXP strain was kindly provided by Dr. Changxu Song. Previously, our group constructed and successfully rescued the recombinant strains rCH/SX/2016-S_HNXP_ (rPEDV-S_wt_) and rCH/SX/2015 (38). PEDV propagation was performed in VERO cells.

### Western blotting

HEK-293T cells seeded in six-well plates were transfected with plasmids expressing full-length S proteins (pCMV6-EV, pCMV6-S_wt_, pCMV6-S_wt-N62A_ [^62^NSTW^65^ to ^62^ASTW^65^ modification], pCMV6-S_mut62_ [^57^ENQGVNST^64^ to ^57^NSSS^60^ modification], pCMV6-S_mutN62A_ [^57^ENQGVNST^64^ to ^57^NSSS^60^ modification, and then ^57^NSSS^60^ to ^57^ASSS^60^ modification], pCMV6-S_mut118_ [^116^NTNA^119^ to ^116^NTSA^119^ modification], pCMV6-S_mut131_ [^131^IKTL^134^ to ^131^NKTL^134^ modification], pCMV6-S_mut235_ [^233^NCIG^236^ to ^233^NCSG^236^ modification], and pCMV6-S_mut722_ [^722^SSTF^725^ to ^722^NSTF^725^ modification]) and truncated proteins (pCMV6-S_D0_, pCMV6-S_D0-N62A_ [^62^NSTW^65^ to ^62^ASTW^65^ modification], pCMV6-S_D0-mut62_ [^57^ENQGVNST^64^ to ^57^NSSS^60^ modification], pCMV6-S_D0-mutN62A_ [^57^ENQGVNST^64^ to ^57^NSSS^60^ modification, and then ^57^NSSS^60^ to ^57^ASSS^60^ modification], pCMV6-S_D0-mut118_ [^116^NTNA^119^ to ^116^NTSA^119^ modification], pCMV6-S_D0-mut131_ [^131^IKTL^134^ to ^131^NKTL^134^ modification], pCMV6-S_D0-mut235_ [^233^NCIG^236^ to ^233^NCSG^236^ modification], pCMV6-S_SD2_, and pCMV6-S_SD2-mut722_ [^722^SSTF^725^ to ^722^NSTF^725^ modification]), respectively. At 36 h post-transfection, the cells were lysed with 200 µL of ice-cold RIPA buffer for 30 min on ice, and then the proteins in the supernatant were collected after centrifugation 12,000 × *g* at 4°C. Lysate aliquots were treated with PNGase F (P0704S; New England BioLabs) according to the manufacturer’s protocols. Western blotting was performed as follows. For the full-length S protein, the samples were separated on a 7% Tris-acetate gel (P0534S, Beyotime) using the BeyoGel Tris-acetate SDS running buffer (P0749, Beyotime) and transferred onto PVDF membranes using western transfer buffer (P0021B, Beyotime). For the truncated S protein, the samples were separated by 12% SDS-PAGE and transferred onto PVDF membranes. The membranes were blocked with 5% nonfat milk in PBST for 2 h at room temperature and then incubated with the PEDV S monoclonal antibody at 4°C overnight. The membranes were washed with PBST and then incubated with goat anti-mouse IgG (H+L) HRP-conjugated secondary antibody, HRP (31430, Thermo Fisher Scientific) for 1 h at room temperature. After washing, the target proteins were detected with an enhanced WesterBright ECL kit (K-12045-D50, Advansta).

### Constructing and rescuing chimeric full-length PEDV cDNA clones

The rPEDV-S_mut62_ (^57^ENQGVNST^64^ to ^57^NSSS^60^ modification), rPEDV-S_mut118_ (^118^NTNA^121^ to ^118^NTSA^121^ modification), rPEDV-S_mut131_ (^131^IKTL^134^ to ^131^NKTL^134^ modification), rPEDV-S_mut235_ (^233^NCIG^236^ to ^233^NCSG^236^ modification), and rPEDV-S_mut722_ (^722^SSTF^725^ to ^722^NSTF^725^ modification) strains were generated using a similar strategy as previously described with some modifications (23). In brief, 2.5 µg of the recombinant BAC plasmids was transfected into VERO cells using Lipofectamine 3000 transfection reagent. These VERO cells were cultured in medium supplemented with trypsin. CPE was monitored daily after transfection. When CPE was evident, the cells and supernatants were collected and subjected to freeze–thaw cycles for passage.

### Identification of the recombinant strains

VERO cells were used for passaging and expansion of the PEDV recombinant strains. The recombinant strains were passaged to the 5th and 10th generations for RNA extraction using RNAiso Plus reagent (TaKaRa, Japan) and reverse transcribed using HiScript II Q RT SuperMix for qPCR (Vazyme, China) according to the manufacturer’s instructions. For sequencing, the fragments were amplified using the corresponding primers, which are listed in Table 3. The recombinant PEDV strains were produced in feces using the same method described above.

**TABLE 3:** Primers in this study^*a*^

Primer name	Sequence (5′−3′)	Usage	Reference
PEDV-S-F1 PEDV-S-R1	AGCCTACCACAAGATGTCAC CTAGTGTCAACACAGAAAGAAC	Sequencing	
PEDV-S-F2	CCATTCAGCGTATTCTTTATTGT	Sequencing	
PEDV-S-R2	CTAAAAGGCAATGCCGCTGC		
PEDV-S-F3	GACTATAAGCGCTGTTCTAATG	Sequencing	
PEDV-S-R3	AGAAGACGCTTTAAACAGTGC		
PEDV-N-F^*b*^	GAATTCCCAAGGGCGAAAAT	RT–qPCR	(35)
N gene probe^*b*^	FAM-CGTAGCAGCTTGCTTCGGACCCA-BHQ		
PEDV-N-R^*b*^	TTTTCGACAAATTCCGCATCT		

^*a*^ F: forward primer. R: reverse primer. FAM, 6-carboxyfluorescein; BHQ, black hole quencher.

^*b*^ Taqman RT-qPCR.

### Indirect IFA

IFA was performed as previously described with slight modifications (23). VERO cells were infected with recombinant viruses for 36 h. The cells were washed with PBS, fixed with 4% paraformaldehyde, and then permeabilized with 0.25% Triton X-100. The cells were blocked with nonfat milk and then incubated with a mouse anti-N monoclonal antibody. The cells were washed with PBS and then incubated with fluorescein isothiocyanate (FITC)-AffiniPure goat anti-mouse IgG (H+L) secondary antibody. Then, the cells were stained with 4-6-diamidino-2-phenylindole (DAPI). Images were acquired with a fluorescence microscope.

### Real-time RT‒qPCR

RNA was extracted from the fecal swab samples using RNAiso Plus reagent and reverse transcribed using HiScript II Q RT SuperMix for qPCR. The cDNA content was determined with the AceQ Universal U+ Probe Master Mix V2 reagent (Vazyme, China) using the primers PEDV-N-F, PEDV-N-R, and the N gene probe shown in Table 3.

### Growth kinetics

VERO cells were infected with recombinant viruses at an MOI of 0.1. After 1 h, the cells were washed with PBS and maintained in the maintenance medium containing 5 µg/mL trypsin. Samples of the cell supernatant were collected at different time points. The viral titer in the supernatants at the indicated time points was determined by the TCID_50_ assay.

### Plaque assay

The plaque assay was performed according to a previous method (38). VERO cells in six-well plates were infected with 10-fold serial dilutions of recombinant PEDVs. After 1 h, the cells were washed with PBS and covered with low-melting agarose containing 5 µg/mL trypsin. The cells were cultured for 4 days, the CPE was observed, and then the plaques were visualized by staining with neutral red dye. The size of the plaque was measured using ImageJ software.

### Evaluation of the pathogenicity of the recombinant strains

Thirty**-**seven newborn piglets negative for transmissible gastroenteritis virus (TGEV), PEDV, PDCoV, and rotavirus (RV) were randomly divided into six groups, with six piglets in each experimental group and seven piglets in the mock group. At 2 days after birth, each piglet was orally inoculated with 1 × 10^4^ TCID_50_ of the recombinant strains or mock inoculated with an equal volume of DMEM. The animals were monitored every 4 h for clinical signs of disease. The severity of diarrhea was scored. Rectal swabs were collected daily, and viral levels in the rectal swab samples were determined. Twelve days after infection, the remaining piglets that did not die or recover from diarrhea were used to calculate the survival rate of each group. Additionally, intestinal tissues obtained from the piglets that died from diarrhea after infection and one piglet in the mock group that was euthanized after 12 days were fixed in 4% paraformaldehyde and stained with H&E. Moreover, IHC staining was also performed using an antibody against the PEDV N protein as the primary antibody. The antigen signals were evaluated using ImageJ software.

### Evaluation of the immunogenicity of the recombinant strains

Twenty-one days after initial infection, blood samples were collected from the surviving piglets. The piglets were subsequently inoculated with the parent strain (rPEDV-S_wt_) at a dose of 10^6^ TCID_50_/pig. The clinical signs and mortality of the piglets were monitored every day. Rectal swabs and throat swabs were collected daily. The number of copies of viral RNA in the rectal swab samples was determined. Blood samples were collected at 7 dpc. All the piglets were euthanized at 9 dpc, and the intestinal tissues were collected for H&E and IHC staining.

### ELISA detection of anti-PEDV N IgG and S lgA in serum, feces, and saliva samples

At 21 dpi/0 dpc and 28 dpi/7 dpc, serum samples were collected. The serum anti-PEDV N IgG and S lgA levels were detected with the ID Screen PEDV Antibody Detection ELISA Kit (IDvet, France) and PEDV lgA Antibody Test Kit (IDEXX, USA) according to the manufacturer’s instructions with slight modifications. About 1 mL of PBS was added to the collected rectal and saliva swabs, which were subsequently centrifuged at 12,000 × *g* for 10 min. SlgA in the feces and saliva was detected using the PEDV lgA Antibody Test Kit (IDEXX, USA) with slight modifications.

### Virus neutralization test

Heat-inactivated sera (56°C for 30 min) were serially diluted fourfold with DMEM. Then, the serum samples were mixed with equal volumes of virus mixture containing 200 TCID_50_ PEDV at 37°C for 90 min. VERO cells were inoculated with the serum–virus mixtures and incubated at 37°C for 1 h. Moreover, incubation with the virus only, medium only, and positive and negative sera were used as controls. After being washed, the cells were cultured with DMEM containing 5 µg/mL trypsin. After 4  days, the neutralizing antibody titer was calculated using the Reed and Muench method. The neutralizing antibody titer of each serum sample was the reciprocal of the dilution with no CPE in 50% of the wells.

### Predictions of protein structures

The N-glycosylation sites were predicted using the NetNGlyc 1.0 server available at https://services.healthtech.dtu.dk/services/NetNGlyc-1.0/. The sequences of PEDV S_wt_, S_mut62_, and S_mut722_ were submitted to the AlphaFold 3 server (39), and the default parameters were used for the S protein trimer structure. Structural analysis and visualization were performed using PyMOL 2.3.0.

### Statistical analysis

The statistical analyses were performed using GraphPad Prism 8.0.2. The data were analyzed using an unpaired *t* test. *P < 0.05, **P < 0.01, and ***P < 0.001 were considered statistically significant. Error bars indicate means ± SDs.

## Data Availability

All data generated during the current study are included in the article.

## References

[1] Zhao Y, Fan B, Song X, Gao J, Guo R, Yi C, He Z, Hu H, Jiang J, Zhao L, Zhong T, Li B. 2024. PEDV-spike-protein-expressing mRNA vaccine protects piglets against PEDV challenge. mBio 15:e0295823. doi:10.1128/mbio.02958-23 38231557 PMC10865985

[2] Tan Y, Sun L, Wang G, Shi Y, Dong W, Fu Y, Fu Z, Chen H, Peng G. 2021. The trypsin-enhanced infection of porcine epidemic diarrhea virus is determined by the S2 subunit of the spike glycoprotein. J Virol 95:e02453-20. doi:10.1128/JVI.02453-20 33692210 PMC8139691

[3] Zhao S, Zhang H, Yang X, Zhang H, Chen Y, Zhan Y, Zhang X, Jiang R, Liu M, Liu L, Chen L, Tang W, Peng C, Gao X, Zhang Z, Shi Z, Gong R. 2021. Identification of potent human neutralizing antibodies against SARS-CoV-2 implications for development of therapeutics and prophylactics. Nat Commun 12:4887. doi:10.1038/s41467-021-25153-x 34373446 PMC8352940

[4] Zhou P, Fan H, Lan T, Yang X-L, Shi W-F, Zhang W, Zhu Y, Zhang Y-W, Xie Q-M, Mani S, et al.. 2018. Fatal swine acute diarrhoea syndrome caused by an HKU2-related coronavirus of bat origin. Nature New Biol 556:255–258. doi:10.1038/s41586-018-0010-9 PMC7094983 29618817

[5] Wrobel AG, Benton DJ, Roustan C, Borg A, Hussain S, Martin SR, Rosenthal PB, Skehel JJ, Gamblin SJ. 2022. Evolution of the SARS-CoV-2 spike protein in the human host. Nat Commun 13:1178. doi:10.1038/s41467-022-28768-w 35246509 PMC8897445

[6] Wang D, Ge X, Chen D, Li J, Cai Y, Deng J, Zhou L, Guo X, Han J, Yang H. 2018. The S gene is necessary but not sufficient for the virulence of porcine epidemic diarrhea virus novel variant strain BJ2011C. J Virol 92:e00603-18. doi:10.1128/JVI.00603-18 29695430 PMC6002738

[7] Hou Y, Meulia T, Gao X, Saif LJ, Wang Q. 2019. Deletion of both the tyrosine-based endocytosis signal and the endoplasmic reticulum retrieval signal in the cytoplasmic tail of spike protein attenuates porcine epidemic diarrhea virus in pigs. J Virol 93:e01758-18. doi:10.1128/JVI.01758-18 30404797 PMC6321913

[8] Hou Y, Ke H, Kim J, Yoo D, Su Y, Boley P, Chepngeno J, Vlasova AN, Saif LJ, Wang Q. 2019. Engineering a live attenuated porcine epidemic diarrhea virus vaccine candidate via inactivation of the viral 2’-o-methyltransferase and the endocytosis signal of the spike protein. J Virol 93:e00406-19. doi:10.1128/JVI.00406-19 31118255 PMC6639265

[9] Hou Y, Lin CM, Yokoyama M, Yount BL, Marthaler D, Douglas AL, Ghimire S, Qin Y, Baric RS, Saif LJ, Wang Q. 2017. Deletion of a 197-amino-acid region in the N-terminal domain of spike protein attenuates porcine epidemic diarrhea virus in piglets. J Virol 91:e00227-17. doi:10.1128/JVI.00227-17 28490591 PMC5487580

[10] Oka T, Saif LJ, Marthaler D, Esseili MA, Meulia T, Lin CM, Vlasova AN, Jung K, Zhang Y, Wang Q. 2014. Cell culture isolation and sequence analysis of genetically diverse US porcine epidemic diarrhea virus strains including a novel strain with a large deletion in the spike gene. Vet Microbiol 173:258–269. doi:10.1016/j.vetmic.2014.08.012 25217400 PMC7126216

[11] Bouhaddou M, Reuschl A-K, Polacco BJ, Thorne LG, Ummadi MR, Ye C, Rosales R, Pelin A, Batra J, Jang GM, et al.. 2023. SARS-CoV-2 variants evolve convergent strategies to remodel the host response. Cell 186:4597–4614. doi:10.1016/j.cell.2023.08.026 37738970 PMC10604369

[12] Johnson BA, Zhou Y, Lokugamage KG, Vu MN, Bopp N, Crocquet-Valdes PA, Kalveram B, Schindewolf C, Liu Y, Scharton D, Plante JA, Xie X, Aguilar P, Weaver SC, Shi PY, Walker DH, Routh AL, Plante KS, Menachery VD. 2022. Nucleocapsid mutations in SARS-CoV-2 augment replication and pathogenesis. PLoS Pathog 18:e1010627. doi:10.1371/journal.ppat.1010627 35728038 PMC9275689

[13] Serman T, Chiang C, Liu G, Sayyad Z, Pandey S, Volcic M, Lee H, Muppala S, Acharya D, Goins C, Stauffer SR, Sparrer KMJ, Gack MU. 2023. Acetylation of the NS3 helicase by KAT5γ is essential for flavivirus replication. Cell Host Microbe 31:1317–1330. doi:10.1016/j.chom.2023.06.013 37478852 PMC10782998

[14] Wu CY, Cheng CW, Kung CC, Liao KS, Jan JT, Ma C, Wong CH. 2022. Glycosite-deleted mRNA of SARS-CoV-2 spike protein as a broad-spectrum vaccine. Proc Natl Acad Sci USA 119:e2119995119. doi:10.1073/pnas.2119995119 35149556 PMC8892489

[15] Yang Q, Hughes TA, Kelkar A, Yu X, Cheng K, Park S, Huang WC, Lovell JF, Neelamegham S. 2020. Inhibition of SARS-CoV-2 viral entry upon blocking N- and O-glycan elaboration. Elife 9:e61552. doi:10.7554/eLife.61552 33103998 PMC7685702

[16] Huang HC, Lai YJ, Liao CC, Yang WF, Huang KB, Lee IJ, Chou WC, Wang SH, Wang LH, Hsu JM, Sun CP, Kuo CT, Wang J, Hsiao TC, Yang PJ, Lee TA, Huang W, Li FA, Shen CY, Lin YL, Tao MH, Li CW. 2021. Targeting conserved N-glycosylation blocks SARS-CoV-2 variant infection in vitro. EBioMedicine 74:103712. doi:10.1016/j.ebiom.2021.103712 34839261 PMC8613501

[17] Zhang S, Liang Q, He X, Zhao C, Ren W, Yang Z, Wang Z, Ding Q, Deng H, Wang T, Zhang L, Wang X. 2022. Loss of Spike N370 glycosylation as an important evolutionary event for the enhanced infectivity of SARS-CoV-2. Cell Res 32:315–318. doi:10.1038/s41422-021-00600-y 35017654 PMC8752327

[18] Liu Y, Wang B, Liang QZ, Shi FS, Ji CM, Yang XL, Yang YL, Qin P, Chen R, Huang YW. 2021. Roles of two major domains of the porcine deltacoronavirus S1 subunit in receptor binding and neutralization. J Virol 95:e0111821. doi:10.1128/JVI.01118-21 34549985 PMC8610578

[19] Li Q, Wu J, Nie J, Zhang L, Hao H, Liu S, Zhao C, Zhang Q, Liu H, Nie L, Qin H, Wang M, Lu Q, Li X, Sun Q, Liu J, Zhang L, Li X, Huang W, Wang Y. 2020. The impact of mutations in SARS-CoV-2 spike on viral infectivity and antigenicity. Cell 182:1284–1294. doi:10.1016/j.cell.2020.07.012 32730807 PMC7366990

[20] Nurdin JA, Kotaki T, Kawagishi T, Sato S, Yamasaki M, Nouda R, Minami S, Kanai Y, Kobayashi T. 2023. N-Glycosylation of rotavirus NSP4 protein affects viral replication and pathogenesis. J Virol 97:e0186122. doi:10.1128/jvi.01861-22 36598201 PMC9888287

[21] Li W, Li H, Liu Y, Pan Y, Deng F, Song Y, Tang X, He Q. 2012. New variants of porcine epidemic diarrhea virus, China, 2011. Emerg Infect Dis 18:1350–1353. doi:10.3201/eid1808.120002 22840964 PMC3414035

[22] Guo J, Fang L, Ye X, Chen J, Xu S, Zhu X, Miao Y, Wang D, Xiao S. 2019. Evolutionary and genotypic analyses of global porcine epidemic diarrhea virus strains. Transbound Emerg Dis 66:111–118. doi:10.1111/tbed.12991 30102851 PMC7168555

[23] Li Z, Ma Z, Han W, Chang C, Li Y, Guo X, Zheng Z, Feng Y, Xu L, Zheng H, Wang X, Xiao S. 2023. Deletion of a 7-amino-acid region in the porcine epidemic diarrhea virus envelope protein induces higher type I and III interferon responses and results in attenuation in vivo. J Virol 97:e0084723. doi:10.1128/jvi.00847-23 37681956 PMC10537754

[24] Zhang H, Zou C, Peng O, Ashraf U, Xu Q, Gong L, Fan B, Zhang Y, Xu Z, Xue C, Wei X, Zhou Q, Tian X, Shen H, Li B, Zhang X, Cao Y. 2023. Global dynamics of porcine enteric coronavirus PEDV epidemiology, evolution, and transmission. Mol Biol Evol 40:msad052. doi:10.1093/molbev/msad052 36869744 PMC10027654

[25] Huang C-Y, Draczkowski P, Wang Y-S, Chang C-Y, Chien Y-C, Cheng Y-H, Wu Y-M, Wang C-H, Chang Y-C, Chang Y-C, Yang T-J, Tsai Y-X, Khoo K-H, Chang H-W, Hsu S-TD. 2022. In situ structure and dynamics of an alphacoronavirus spike protein by cryo-ET and cryo-EM. Nat Commun 13:4877. doi:10.1038/s41467-022-32588-3 35986008 PMC9388967

[26] Diep NV, Norimine J, Sueyoshi M, Lan NT, Yamaguchi R. 2017. Novel porcine epidemic diarrhea virus (PEDV) variants with large deletions in the spike (S) gene coexist with PEDV strains possessing an intact S gene in domestic pigs in Japan: a new disease situation. PLoS One 12:e0170126. doi:10.1371/journal.pone.0170126 28095455 PMC5241010

[27] Tsai KJ, Deng MC, Wang FI, Tsai SH, Chang C, Chang CY, Huang YL. 2020. Deletion in the S1 region of porcine epidemic diarrhea virus reduces the virulence and influences the virus-neutralizing activity of the antibody induced. Viruses 12:1378. doi:10.3390/v12121378 33276502 PMC7761297

[28] Li C, Li W, Lucio de Esesarte E, Guo H, van den Elzen P, Aarts E, van den Born E, Rottier PJM, Bosch B-J. 2017. Cell attachment domains of the porcine epidemic diarrhea virus spike protein are key targets of neutralizing antibodies. J Virol 91:e00273-17. doi:10.1128/JVI.00273-17 28381581 PMC5446644

[29] Chang CY, Cheng IC, Chang YC, Tsai PS, Lai SY, Huang YL, Jeng CR, Pang VF, Chang HW. 2019. Identification of neutralizing monoclonal antibodies targeting novel conformational epitopes of the porcine epidemic diarrhoea virus spike protein. Sci Rep 9:2529. doi:10.1038/s41598-019-39844-5 30792462 PMC6385244

[30] Zhang G, Peng Q, Liu S, Fan B, Wang C, Song X, Cao Q, Li C, Xu H, Lu H, Bao M, Yang S, Li Y, Wang J, Li B. 2024. The glycosylation sites in RBD of spike protein attenuate the immunogenicity of PEDV AH2012/12. Virus Res 345:199381. doi:10.1016/j.virusres.2024.199381 38679392 PMC11070342

[31] Williams JA, Biancucci M, Lessen L, Tian S, Balsaraf A, Chen L, Chesterman C, Maruggi G, Vandepaer S, Huang Y, Mallett CP, Steff AM, Bottomley MJ, Malito E, Wahome N, Harshbarger WD. 2023. Structural and computational design of a SARS-CoV-2 spike antigen with improved expression and immunogenicity. Sci Adv 9:eadg0330. doi:10.1126/sciadv.adg0330 37285422 PMC10246912

[32] Xu Y, Yan X, Wei T, Chen M, Zhu J, Gao J, Liu B, Zhu W, Liu Z. 2023. Transmucosal delivery of nasal nanovaccines enhancing mucosal and systemic immunity. Nano Lett 23:10522–10531. doi:10.1021/acs.nanolett.3c03419 37943583

[33] Chattha KS, Roth JA, Saif LJ. 2015. Strategies for design and application of enteric viral vaccines. Annu Rev Anim Biosci 3:375–395. doi:10.1146/annurev-animal-022114-111038 25387111

[34] Yuan C, Zhang P, Liu P, Li Y, Li J, Zhang E, Jin Y, Yang Q. 2022. A novel pathway for porcine epidemic diarrhea virus transmission from sows to neonatal piglets mediated by colostrum. J Virol 96:e0047722. doi:10.1128/jvi.00477-22 35758666 PMC9327711

[35] Deng X, Buckley AC, Pillatzki A, Lager KM, Faaberg KS, Baker SC. 2020. Inactivating three interferon antagonists attenuates pathogenesis of an enteric coronavirus. J Virol 94:e00565-20. doi:10.1128/JVI.00565-20 32554697 PMC7431798

[36] Jiao Y, Zhao P, Xu LD, Yu JQ, Cai HL, Zhang C, Tong C, Yang YL, Xu P, Sun Q, Chen N, Wang B, Huang YW. 2024. Enteric coronavirus nsp2 is a virulence determinant that recruits NBR1 for autophagic targeting of TBK1 to diminish the innate immune response. Autophagy 20:1762–1779. doi:10.1080/15548627.2024.2340420 38597182 PMC11262224

[37] Lu Y, Cai H, Lu M, Ma Y, Li A, Gao Y, Zhou J, Gu H, Li J, Gu J. 2020. Porcine epidemic diarrhea virus deficient in RNA cap guanine-N-7 methylation is attenuated and induces higher type I and III interferon responses. J Virol 94:e00447-20. doi:10.1128/JVI.00447-20 32461321 PMC7394890

[38] Li Z, Ma Z, Dong L, Yang T, Li Y, Jiao D, Han W, Zheng H, Xiao S. 2022. Molecular mechanism of porcine epidemic diarrhea virus cell tropism. MBio 13:e0373921. doi:10.1128/mbio.03739-21 35285698 PMC9040822

[39] Abramson J, Adler J, Dunger J, Evans R, Green T, Pritzel A, Ronneberger O, Willmore L, Ballard AJ, Bambrick J, et al.. 2024. Accurate structure prediction of biomolecular interactions with AlphaFold 3. Nature 630:493–500. doi:10.1038/s41586-024-07487-w 38718835 PMC11168924

